# Sediment Delivery Ratio of Single Flood Events and the Influencing Factors in a Headwater Basin of the Chinese Loess Plateau

**DOI:** 10.1371/journal.pone.0112594

**Published:** 2014-11-12

**Authors:** Mingguo Zheng, Yishan Liao, Jijun He

**Affiliations:** 1 Key Laboratory of Water Cycle and Related Land Surface Processes, Institute of Geographic Sciences & Natural Resources Research, Chinese Academy of Sciences, Beijing, China; 2 Guangdong Institute of Eco-environment and Soil Sciences, Guangzhou, China; 3 Base of the State Laboratory of Urban Environmental Processes and Digital Modelling, Capital Normal University, Beijing, China; Centro de Investigacion Cientifica y Educacion Superior de Ensenada, Mexico

## Abstract

Little is known about the sediment delivery of single flood events although it has been well known that the sediment delivery ratio at the inter-annual time scale is close to 1 in the Chinese Loess Plateau. This study examined the sediment delivery of single flood events and the influencing factors in a headwater basin of the Loess Plateau, where hyperconcentrated flows are dominant. Data observed from plot to subwatershed over the period from 1959 to 1969 were presented. Sediment delivery ratio of a single event (*SDR*_e_) was calculated as the ratio of sediment output from the subwatershed to sediment input into the channel. It was found that *SDR*_e_ varies greatly for small events (runoff depth <5 mm or rainfall depth <30 mm) and remains fairly constant (approximately between 1.1 and 1.3) for large events (runoff depth >5 mm or rainfall depth >30 mm). We examined 11 factors of rainfall (rainfall amount, rainfall intensity, rainfall kinetic energy, rainfall erosivity and rainfall duration), flood (area-specific sediment yield, runoff depth, peak flow discharge, peak sediment concentration and flood duration) and antecedent land surface (antecedent precipitation) in relation to *SDR*_e_. Only the peak sediment concentration significantly correlates with *SDR*_e_. Contrary to popular belief, channel scour tends to occur in cases of higher peak sediment concentrations. Because small events also have chances to attain a high sediment concentration, many small events (rainfall depth <20 mm) are characterized by channel scour with an *SDR*_e_ larger than 1. Such observations can be related to hyperconcentrated flows, which behave quite differently from normal stream flows. Our finding that large events have a nearly constant *SDR*_e_ is useful for sediment yield predictions in the Loess Plateau and other regions where hyperconcentrated flows are well developed.

## Introduction

Sediment yield represents the total quantity of sediment observed at a certain point in a landscape or a river system, such as the watershed outlet, in a specified time interval. The sediment yield prediction is of key interest in stream and watershed management due to increasing concerns on water quality, aquatic habitat, biodiversity and life of man-made structures (dams, bridges, harbors, and water supply systems) [1], [2]. The concept of sediment delivery ratio (SDR), commonly defined as the ratio of sediment yield to gross erosion [3], [4], provides a convenient way to estimate sediment yield to a point of interest. In equation form *SDR* is expressed as
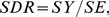
(1)where *SY* (mass per unit time) represents sediment yield at a point of interest, and *SE* (mass per unit time) represents gross erosion rate of the area upstream of that point. It is believed that if the relationship between SDR and its influential factors is well established, equation (1) would be greatly helpful for estimating sediment yields in ungauged locations. Because of the simplicity in concept and the ability to link on-site erosion with downstream sediment yield, studies of SDR have received much attention [4]–[6]. SDR is affected by fluvial processes operating at a variety of spatial scales from slopes to channels. Factors affecting SDR almost includes all variables representing hydrological regime (e.g. flood and rainfall) and watershed prosperities (e.g. topography, vegetation and land use). Due to the multitude of the influencing factors and their interactions, it is difficult to identify the dominant controls on SDR [7]–[9]. As a result, the established relationships between SDR and the influencing factors are largely empirical and can hardly extrapolate beyond the data range with confidence [7]–[10].

The Chinese Loess Plateau is famous for its high-intensity soil erosion, which frequently exceeds 10, 000 t km^−2^ a^−1^. Gong and Xiong [11] proposed that SDR is as high as 1 in the Loess Plateau. Mou and Meng [12] subsequently found that almost all sediments (>95%) are moved as wash load as a result of the fine texture of the loess in combination with the strong sediment transport capacity [13] of hyperconcentrated flows, which are well developed in the Loess Plateau [14] and behave quite differently from normal sediment-laden streamflow [15]–[17]; this mechanism physically enables a SDR as high as 1. Nowadays, it has been widely accepted that SDR in the Loess Plateau is close to 1 over a wide range of basin sizes at inter-annual time scale [5], [7], [14], [18]–[20]. Nevertheless, knowledge of the sediment delivery and the influencing factors is currently lacking at the time scale of the flood event in the loess Plateau. Among more than 100 rainstorm events over a single year, only 2–7 rainstorm events are erosive in the Loess Plateau [21]. Research efforts are, therefore, needed to investigate sediment delivery processes at the event time scale.

**Figure 1:**
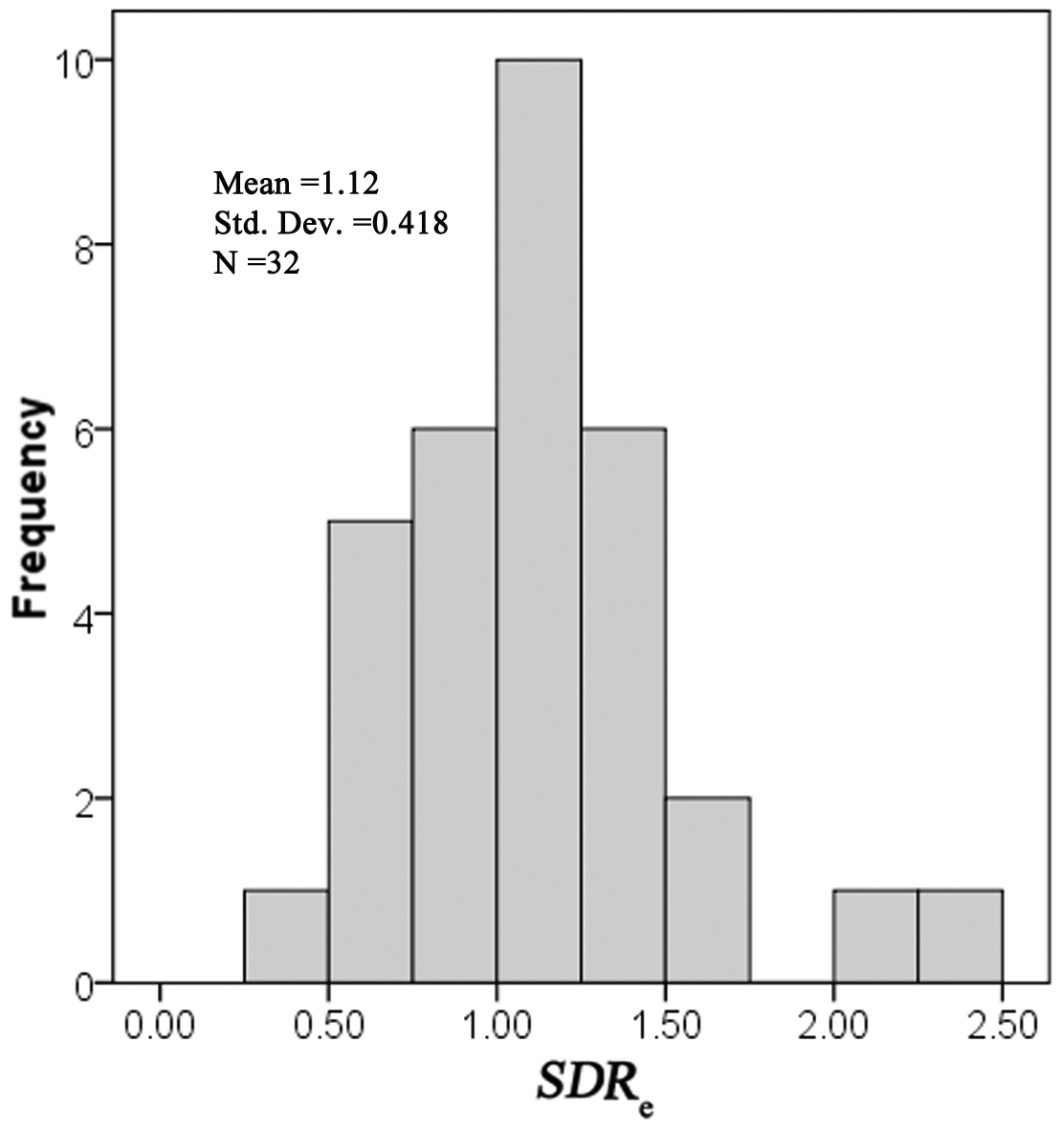
Histograms of *SDR*_e_ for 32 flood events observed at the Tuanshangou station.

One of great concerns in determining SDR is the enormous uncertainty introduced by estimating gross erosion [1], [3], i.e. the denominator in Equation (1). To guarantee a reasonable estimation of the gross erosion and in turn, the SDR, we limited our study to the Tuanshangou subwatershed (Fig. 1), a headwater basin of the first-order channel in the Loess Plateau. The subwatersheds, where eroded sediments are primarily sourced, are the endmember unit for soil conservation practices in the Loess Plateau. The object of this study is to examine sediment delivery processes of single events in the Tuanshangou subwatershed, hoping to further the knowledge of fluvial processes under the control of hyperconcentrated flows. We firstly calculated the SDR for single events and then, examined a number of factors in relation to sediment delivery, including factors of rainfall, flood and antecedent land surface.

## Study Area and Data Source

The Tuanshangou Creek (latitude 37°41′N, longitude 109°58′E; See Fig. 1 in [22] for the location) drains an area of 0.18 km^2^. Typical of the Loess Plateau, the local loess mantle is thicker than 100 m. As wind-borne dust in Quaternary times, loess is loosely compact and highly erodible. The climate is typically semi-arid. During the monitoring period (1959–1969), the mean average annual precipitation is approximately 450 mm and the maximum 30-min rainfall intensity is as high as 2.17 mm min^−1^. The mean slope of the Tuanshangou subwatershed is as high as 26.8°. The valley side slope is particularly steep (>35°), allowing active mass wasting such as slumping, sliding and collapsing. Approximately 80% of the area was under arable without soil conservation practices. Other lands were abandoned due to the precipitous topography.

Observations at the subwatershed outlet (i.e. the Tuanshangou station) show that the annual sediment yield varied from 200 to 72 000 t km^−2^ a^−1^ with a mean of 19 700 t km^−2^ a^−1^ during the monitoring period. The instantaneous sediment concentrations of storm runoff frequently exceeded 1 000 kg m^−3^ and the mean sediment concentration of flood events was 742 kg m^−3^ (See Table 4 in [22]), much higher than the concentration threshold between the normal sediment-laden flow and the hyperconcentrated flow in the Loess Plateau (200 kg m^−3^ [23] or 300–400 kg m^−3^ [14]).

## Data and Methods

### Data

Unless stated otherwise, all data used were obtained from the Yellow River Water Conservancy Commission (YRWCC). The YRWCC stream-gauging crews conducted all measurements following national standard procedures of China [24], which have been described in [22] and [25].

This study primarily used data observed at three experimental sites: the Tuanshangou station and two runoff plots within the Tuanshangou subwatershed (i.e. Plots 7 and 9 in [22] and [25]). Both plots were under arable. Crops varied between years, including millet, potato, mung bean, clover, sorghum and wheat. The plots are composed of two parts: hill slope and valley side slope. Such slopes are conventionally termed as the entire slope in Chinese literatures. The plot lengths were 126 m and 164 m, respectively. Detailed information of the plots is available in Table 2 in [22]. Hyetograph data were obtained using a rainfall gauge near Plot 7.

### Calculations of SDR

This study defined the gross erosion, i.e. the denominator in Equation (1), as sediment input into stream channels, as did in [18], [26]. Such a definition in effect reflects the sediment transport efficiency of stream channels [27]. Plots 7 and 9 were large enough for gullies to develop, through which overland flows drain into the Tuanshangou Creek (See Fig. 2 in [25]). Plot 7 was in the lower part and Plot 9 was in the upper part of the Tuanshangou Creek. Hence, we can use the average of the collective discharges of sediment at Plots 7 and 9 to estimate the sediment input into the creek. We calculated SDR of a single flood event (*SDR*_e_, dimensionless) as follows:
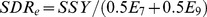
(2)where *SSY* (t km^−2^) represents observed area-specific sediment yield at the Tuanshangou station, and *E*_7_ and *E*_9_ (t km^−2^) represents erosion intensity at Plots 7 and 9 for a rainfall event, respectively. Comparisons between *E*_7_ and *E*_9_ on the same rainfall day produced a regression coefficient very close to 1 (0.94) and a *R* ^2^ of 0.93, suggesting that the erosion intensity may not vary greatly among entire slopes within the Tuanshangou subwatershed. We thus believe that the average of *E*_7_ and *E*_9_, i.e. the denominator of Equation (2), reasonably represents the sediment discharge into the Tuanshangou Creek per unit area.

The *SDR*_e_ values obtained from Equation (2) can be larger or smaller than 1. A *SDR*_e_ larger than 1 indicates channel degradation, while a *SDR*_e_ smaller than 1 indicates channel aggradation. When *SDR*_e_ is equal to 1, stream channels are in equilibrium.

### Factors influencing sediment delivery

Factors influencing sediment delivery can be grouped into three categories: rainfall factors, flood factors and antecedent land surface factors. SDR is related to not only flow discharge but also the rheologic and fluid proprieties of flows, which largely depends on suspended load in flows. Flood factors we examined thus include five factors: *SSY* (t km^−2^), *h* (runoff depth of a flood event, mm), *q*_max_ (peak flow discharge of a flood event, m^3^ s^−1^), *C*_max_ (maximum sediment concentration of a flood event, kg m^−3^) and *T*_f_ (flood duration, min). All flood factors were measured at the Tuanshangou station.

Rainfall factors we examined also included 5 factors: *P* (rainfall depth, mm), *I*_30_ (the maximum 30-min rainfall intensity, mm min^−1^), *E* (rainfall kinetic energy, J m^−2^), *EI*_30_ (the product of *E* and *I*_30_) and *T*_p_ (rainfall duration, min). *EI*_30_, the rainfall erosivity index of the Universal Soil Loss Equation (USLE) [28], is the most common rainfall erosivity index. *E* was calculated using the following relationship:
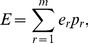
(3)where *e_r_* is the rainfall kinetic energy per unit depth of rainfall per unit area (J m^−2^ mm^−1^), and *p_r_* is the depth of rainfall (mm) for the *r*th interval among *m* intervals of the storm hyetograph. *e_r_* is calculated using an empirical equation for the Loess Plateau [29]:
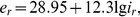
(4)where *i_r_* (mm min^−1^) represents the mean rainfall intensity for the *r*th interval. Equation (4), built on measurements of the drop size distribution of 195 storms, is almost identical to the rainfall intensity-energy equation of the USLE [28] after unit conversion.

Pre-event factors we examined only includes the antecedent precipitation index (*P′*, mm), which is a surrogate for pre-event soil moisture and an important factor affecting runoff yield and soil erodibilities [30]. The vegetation cover rarely exceeded 25% in the Tuanshangou subwatershed. Hence, we do not take the vegetation cover into consideration. *P′* is defined as [31], [32]:
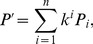
(5)where *n* is the number of antecedent days, *P_i_* (mm) is the daily precipitation for the *i*th day prior to the event, and *k* (dimensionless) is the decay constant representing the outflow of the regolith. In practices, *k* generally lie between 0.80 and 0.98 and *n* is typically 5, 7 or 14 days [31], [33]. Here, *k* is set at 0.9 following [34]. The gauging crews made measurements of soil moisture near Plot 7. The correlation between *P′* and the observed soil moisture (top 30 cm) increases asymptotically with increasing *n*. Because the correlation varies little when *n* exceeds 11 days [35], *n* is taken as 11 days in this study. The resultant *P′* is well correlated with the observed soil moisture (*r* = 0.85, *p*<0.001).

## Results and Discussion

### The calculation results of SDR

A total of 36 storm events were well monitored simultaneously at the three sites: the Tuanshangou station and Plots 7 and 9. We calculated *SDR*_e_ for all of the events. The bedrock is exposed at the channel bed of the Tuanshangou Creek. Because the bedrock is more prone to runoff production than loess slopes, runoff and sediment are primarily sourced from the channel bed in cases of small rainfall intensities. Among 11 small events with *h* smaller than 1 mm, four had a *SDR*_e_ (2.8, 5.7, 6.3 and 23.7 respectively) distinctively higher than others (the maximum is 1.2), implying that these events essentially conveyed pre-event sediment storage on the channel bed rather than soils eroded from uplands. The four events all had a runoff depth not greater than 0.1 mm at Plots 7 and 9, implying that the sediment discharge into the channel was small enough to be neglected. We removed the four events from the subsequent analyses and the subsequent calculation of *SDR*_e_ involves 32 events.

**Figure 2:**
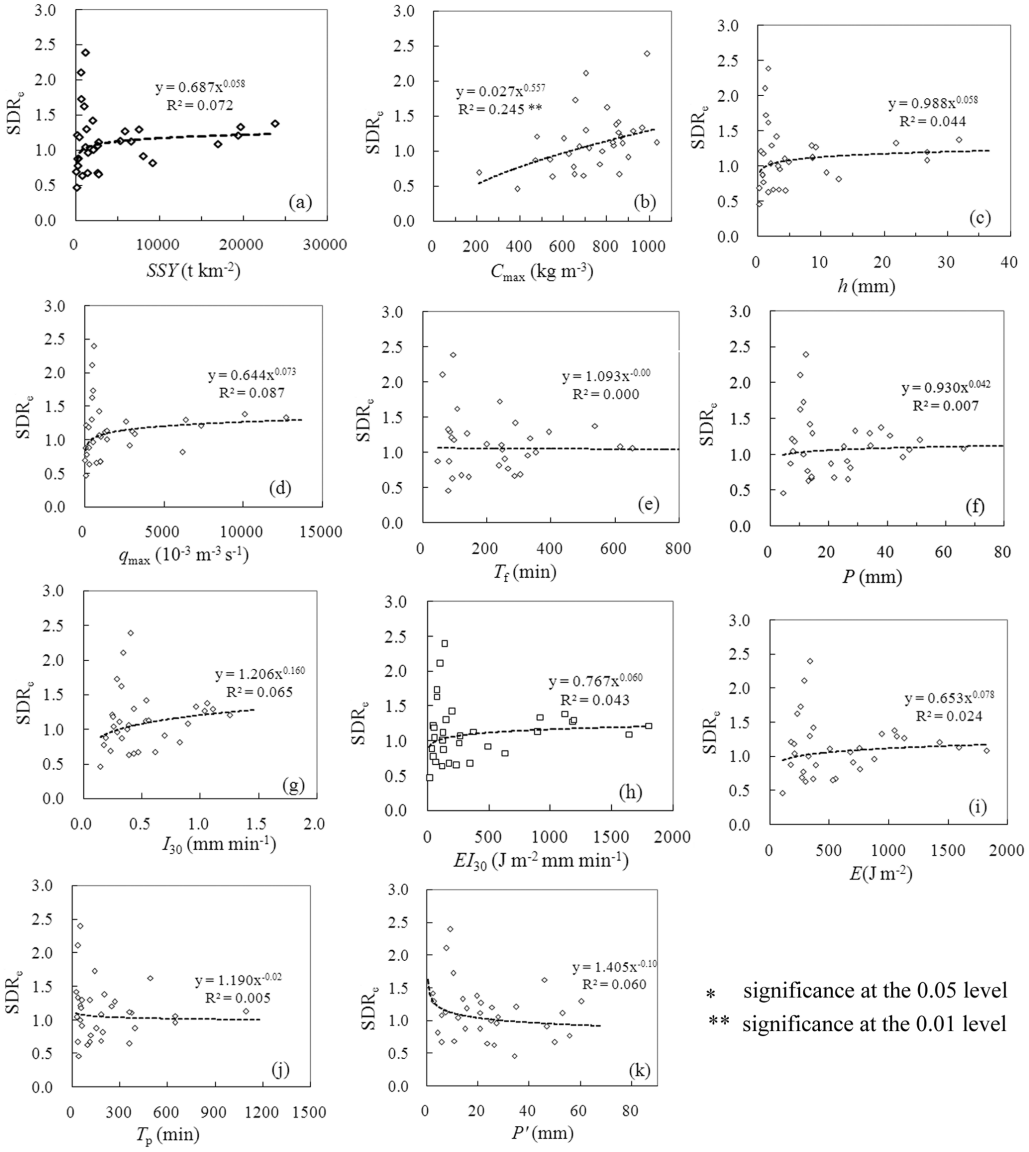
*SDR*_e_ in relation to flood factors (a–e), rainfall factors (f–j) and the pre-event factor (k).

Histograms of *SDR*_e_ for the 32 events are presented in Fig. 1. *SDR*_e_ ranges from 0.46 to 2.39 with a mean of 1.12 and a median of 1.1. Twenty events have a *SDR*_e_ larger than 1. As shown in Fig. 2, *SDR*_e_ varies greatly for small events (approximately *h*<5 mm or *P*<30 mm) and remains fairly constant for large events (*h*>5 mm or *P*>30 mm). Only in cases of small events can significant channel degradation or aggradation occurs. For major sediment-producing events (*SSY*>5000 t km^−2^, *h*>5 mm or *P*>30 mm), *SDR*_e_ essentially lies between 1.1–1.3 (Fig. 2(a), (c) and (f)). Interesting to note is that many small events (*P*<20 mm; Fig. 2(f)) have a *SDR*_e_ much larger than 1.

### Flood factors in relation to sediment delivery

Among 11 factors we examined, only *C*_max_ are significantly correlated with *SDR*_e_ (*p* = 0.004; Fig. 2). Nevertheless, the considerable scatters, as shown in Fig. 2(b), prevent *C*_max_ from being a good predictor of *SDR*_e_.

Contrary to popular belief, *SDR*_e_ increase with increasing *C*_max_ (Fig. 2(b)), a phenomenon also reported in [18]. When *C*_max_ is higher than 700 kg m^−3^, most of the events (16 out of 19) have a *SDR*_e_ larger than 1, indicating channel scour. In contrast, most of the events (9 out of 13) correspond to a *SDR*_e_ smaller than 1 when *C*_max_ is smaller than 700 kg m^−3^, indicating channel fill. This observation can be related with hyperconcentrated flows. Different from normal sediment-laden flows, the energy expenditure on suspended-load motion of hyperconcentrated flows decreases with increasing sediment load, as evidenced by laboratory experiments and field observations [14], [17], [36]. As a result, the sediment transport capacity of hyperconcentrated flows would increase with sediment concentrations and thus, the channel scour is more likely to occur at high rather than at small *C*_max_, as has also been observed in the main stream of the Yellow River (See Fig. 3 in [14]).

Sediment delivery along a stream channel depends on not only sediment transport capacity of flows, but also sediment availability within channels. The time interval between large flood events, which occurs relatively infrequently, is generally longer than small ones. Numerous preceding runoff events can prepare a large amount of sediment storage within channels prior to a large flood event. Mass wasting also occurs more readily during large events. Hence, large flood events generally have a *SDR*_e_ larger than 1.

**Figure 3:**
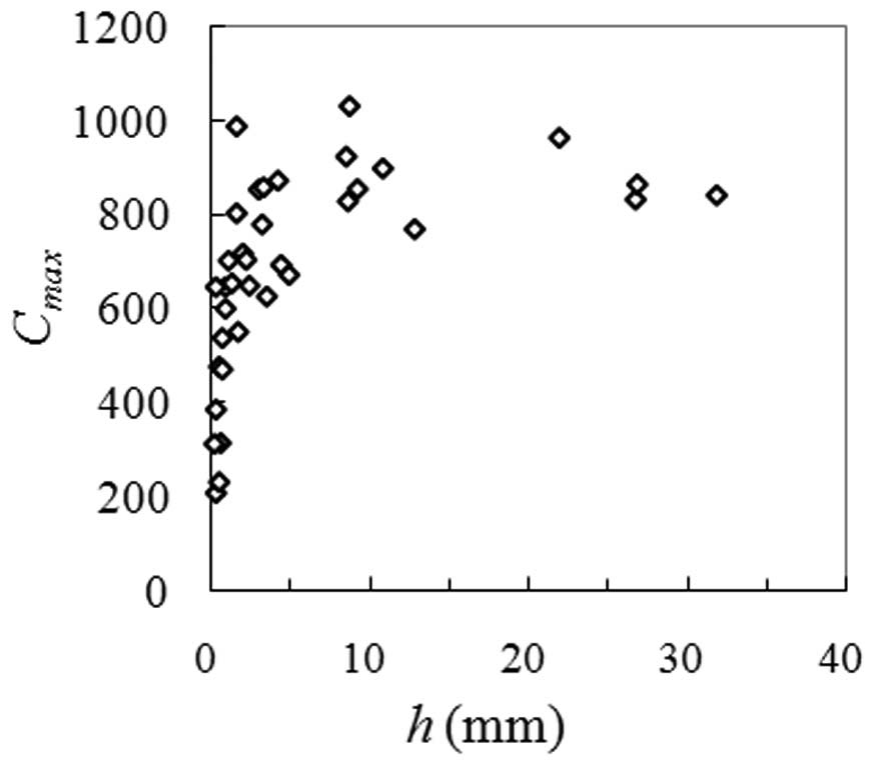
The relationship between *C*_max_ and *h* at the Tuanshangou station, showing that small runoff events also have chances to achieve a high level of sediment concentration.

As indicated in Fig. 2(a), (c) and (d), *SDR*_e_ is larger than 1 not only for large events but also for many small events, as opposed to that observed in the Murray Darling Basin of Australia [7]. No direct relationship exists between sediment concentration and water discharge for hyperconcentrated flows [13]. Small events also have chances to attain a high level of sediment concentration (Fig. 3) and thus, a high sediment transport capacity. In addition, antecedent sediment storage within channels may contribute a large part of the event sediment yield considering the minuscule sediment yield of a small event. In contrast, antecedent sediment storage within channels can hardly form the major sediment source for large events due to their tremendous sediment yields. Only for small events, hence, can *SDR*_e_ be distinctively greater than 1. In contrast, *SDR*_e_ for large events falls within a narrow range between approximately 1.1 and 1.3.

### Rainfall factors and antecedent precipitation in relation to sediment delivery

None of Rainfall properties show correlation with *SDR*_e_ (Fig. 2(f)–(j)). Raindrops splash soil particles and provide sediment for overland flows. Rainfall impact also increases the turbulence of sheet flow and thus, enhances its ability to detach soil and to transport sediment. In both ways, rainfall exerts direct effects on sediment delivery. However, rill erosion and gully erosion are strongly dominant over splash erosion in the Loess Plateau [37], [38]. Meanwhile, raindrop impact has no effect on rill flows or other concentrated flows because the turbulent effect of raindrop impact is attenuated with increasing water depth [39]–[41]. Consequently, the both direct mechanisms that rainfall affects sediment delivery become ineffective. Though the large rainfall event corresponds to a high *q*_max_ resulting a high sediment transport capacity, the small event can achieve a high transport capacity by achieving a high sediment concentration. Consequently, the indirect mechanism also poses no impact on *SDR*_e_.

For the same reason as above, it cannot be expected that a high antecedent soil moisture results in a high *SDR*_e_ by increasing *q*_max_. Due to high vulnerability to water erosion at high antecedent soil moistures, sediment concentration and thus sediment transport capacity of hyperconcentrated flows may be enhanced. However, this enhancement should be obscured in a site where mass wasting events are active. Small-sized mass wasting events, such as bank failure and knickpoint retreat, even act as an important agent for rill development [42]–[44]. Indeed, there is no correlation between *P′* and *C*_max_ (*p* = 0.37). As a result, *SDR*_e_ are totally independent of *P′* (Fig. 2(k)). Similarly, due to intensive mass wastings in the Loess Plateau, vegetation and slope land measures for soil conservation, such as terraces and ridges, have no effect on sediment concentrations at the watershed outlet [45].

## Conclusions

This study calculated the sediment delivery ratio of single flood events (*SDR*_e_) and examined the factors of rainfall, flood and antecedent land surface in relation to *SDR*_e_ in a headwater basin of the Loess Plateau, where hyperconcentrated flows dominate the fluvial processes. *SDR*_e_ were calculated as the ratio of sediment output from the subwatershed to sediment input into the channel. Due to distinct behaviours of hyperconcentrated flows, the sediment delivery process of the examined subwatershed is quite different from that under the control of the normal stream flow:

1. *SDR*_e_ varies greatly for small events (*h*<5 mm or *P*<30 mm) and remains fairly constant for large events (*h*>5 mm or *P*>30 mm). Most of the examined events (20 out of 32) have a *SDR*_e_ higher than 1, implying channel degradation. Such high sediment transfer efficiency can be related to hyperconcentrated flows, which have very strong capacity to transport sediment.
2. Due to decreasing energy expenditure on suspended-load motion with increasing sediment load for hyperconcentrated flows, *SDR*_e_ show increasing trends with the increasing level of sediment concentration, as indexed by the maximum sediment concentration of a flood event (*C*_max_). Channel degradation primarily occurs when *C*_max_ exceed 700 kg m^−3^. Otherwise, channel aggradation occurs. Among 11 factors we examined, only *C*_max_ is correlated with *SDR*_e_ (*p*<0.01).
3. Small events also have chances to attain a high *C*_max_, thereby leading to a *SDR*_e_ higher than 1. Moreover, the extremely large *SDR*_e_ always corresponds to small events because pre-event sediment storage within channels can hardly form the major sediment source for large events. Because both large and small events are capable of achieve a high *SDR*_e_, the peak flow discharge is poorly correlated with *SDR*_e_.
4. Both rainfall factors (including rainfall amount, rainfall intensity, rainfall kinetic energy, rainfall erosivity and rainfall duration) and antecedent precipitation show no correlation with *SDR*_e_. Due to poor correlations and considerable scatters, any factors we examined cannot be expected to be a good predictor of *SDR*_e_. Nevertheless, our finding that large flood events (*h*>5 mm or *P*>30 mm) has similar values of *SDR*_e_ in a narrow range between approximately 1.1 and 1.3 should be a valuable aid to the sediment yield prediction in the Loess Plateau given the fact that large events contribute almost all sediments.
